# The Heterogeneous Effects of Formal and Informal Environmental Regulation on Green Technology Innovation—An Empirical Study of 284 Cities in China

**DOI:** 10.3390/ijerph20021621

**Published:** 2023-01-16

**Authors:** Chuantang Ren, Tao Wang, Yue Wang, Yizhen Zhang, Luwei Wang

**Affiliations:** School of Geography, Nanjing Normal University, Nanjing 210023, China

**Keywords:** green technology innovation, formal environmental regulation, informal environmental regulation, spatial and urban heterogeneity, Chinese cities

## Abstract

Promoting green technology innovation (GTI) through environmental regulation is a key measure in reducing the severity of environmental problems. However, the effects of formal environmental regulation (FER) and informal environmental regulation (IER) on GTI have not been clarified. Through theoretical analysis, this paper analyzes the effects of FER and IER on GTI based on OLS and GTWR models. The results show the following: (1) In all Chinese cities, both FER and IER have had a positive impact on GTI. The impact of FER has been much stronger than that of IER. They show a linkage effect, and their interaction (TER) has had a positive impact on GTI. (2) In terms of spatial heterogeneity, the impact of FER, IER, and TER on GTI has decreased across the east–west gradient and has been supplemented by a core–periphery structure. (3) In terms of urban heterogeneity, the impact of FER, IER, and TER has decreased with the size of the city. This study has the potential to strengthen the effect of environmental regulation on GTI. It can provide a decision-making reference for cities to coordinate FER and IER strategies, and provides evidence for adopting regionally differentiated environmental regulation strategies.

## 1. Introduction

Since 1978, China has achieved an industrialization catch-up and an economic take-off under a development model of competitive growth. However, this long-term extensive economic growth model has caused such problems as an excessive consumption of resources and environmental pollution. The traditional resource-consuming industrial development model was contrary to the concept of sustainable development, and the transformation into a green and healthy development model has become an urgent issue. With the proposal and advancement of the concept of an ecological civilization, China’s government has attached great importance to saving resources and protecting the environment. For example, China’s government has put forward Five Development Concepts: Innovation, Coordination, Greenness, Openness, and Sharing. Recently, China’s 14th Five-Year Plan put forward a two-carbon strategy of “striving to reach a carbon peak by 2030 and achieving carbon neutrality by 2060” to promote green development to a new height. Against this background, the transformation of green technology to promote environmental protection has become the key to sustainable development [1]. However, due to the positive externalities of environmental protection and green technology innovation, the spontaneous regulation of the market has failed. Therefore, environmental regulation has become an important way to stimulate energy conservation, emission reduction and green technology innovation [2].

The relationship between environmental regulation (ER) and green technology innovation (GTI) has become the focus of attention in many fields such as regional economics, urban economics, and environmental economics [3,4,5]. Comprehensive analysis of whether ER can promote GTI shows the following: (1) ER has a negative impact on GTI. Neoclassical economists have put forward the Compliance Cost Hypothesis, which assumes that the technology, resource allocation and demand of enterprises are already in a steady state before environmental regulations are implemented. Environmental regulations will increase production costs for enterprises, distort the allocation of factors, crowd out innovation investment, and finally lead to a decline in their ability to make green technology innovations [6,7,8]. Numerous studies have been empirically tested with multiple industries in the United States, the United Kingdom, Germany, and other countries [7,9,10,11]. These studies found that ER can lead to higher costs for enterprises and damage enterprises’ productivity and innovation capabilities [12]. These researches were based on the theory of Compliance Cost Hypothesis and emphasized the short-term cost increase caused by environmental regulation to enterprises, ignoring the independent initiative and long-term innovation benefits of enterprises in response to environmental regulation. (2) ER has a positive impact on GTI. The Porter Hypothesis posits the view that ER can promote GTI. Environmental regulations can force enterprises to engage in green technology innovation, which helps them to increase productivity and create an “innovation offset effect” while achieving environmental protection goals [13,14,15,16]. Many studies have also confirmed this view through an analysis of multiple countries [17,18,19,20,21,22]. These researches were based on the Porter Hypothesis, i.e., that enterprises can achieve green innovation in response to environmental regulations, and idealized the complex behavior of enterprises in response to environmental regulations as green innovation, which was inconsistent with the reality. (3) The effect of ER on GTI is complex. Some studies have pointed out that the mechanism of ER on GTI is complex and cannot be generalized. The specific performance is in terms of time, space, the nature of the enterprise, the characteristics of the industry, the type of technology, and the uncertainty of action conditions [23,24,25]. For example, many studies have found that the relationship between ER and GTI is unclear, or that the effect is nonlinear, temporally heterogeneous, and regionally heterogeneous [26,27,28,29,30,31]. On the whole, these researches are too limited in content, and most of them are aimed at a certain region or industry. The conclusions drawn are difficult to promote, and the references of the research conclusions are limited.

From the perspective of the type of environmental regulation, early studies focused on formal environmental regulation (FER), which is a government-led type of regulation that imposes mandatory pollution control measures on polluting enterprises [32]. As public awareness of environmental protection has increased, the effect of public-oriented environmental regulation has gradually become prominent [33]. In 1996, scholars first defined it as informal environmental regulation (IER) [34]. As research progresses, scholars have studied the relationship between IER and environmental pollution [35,36]. In contrast, relatively little research has been done on IER, and the effect of IER on GTI needs to be further studied [30,37]. Since enterprises are the main culprit of environmental pollution and the main sources of technological innovation, green technological innovation is generally considered to be the result of enterprises responding to environmental regulations [38,39,40]. Therefore, studies on enterprises have become mainstream, while investigations on the city need to be further conducted. Due to the regional differences in China, decentralization leads to local competition, so China has become an ideal case study for this topic [41]. A review of the research showed that how GTI is effectively stimulated by ER has become a key issue that needs to be solved urgently in the construction of environmental policy systems at this stage. However, there are some gaps in the existing research on ER and GTI. First, the research on IER is relatively weak, and the existing research is mostly focused on the effect of FER. Specifically, the effect of IER on GTI and its similarities and differences with FER need to be further studied. In addition, China has a vast territory and considerable differences in development between the east, center and west. At the same time, the sizes of the cities vary greatly in China. The study of the spatial heterogeneity and urban heterogeneity of GTI by FER and IER is an obvious gap. Considering these gaps, this paper provides an analysis based on the Compliance Cost Hypothesis and Porter Hypothesis and considers China as a case for empirical research. First, this paper focuses on the effect of IER on GTI and compares the effect of IER with that of FER. Second, this paper analyzes the spatial and urban heterogeneity of the influence of FER and IER on GTI, contributing to the improvement of the spatial and urban heterogeneity of environmental regulation. This study can provide a reference for the implementation of differentiated environmental regulatory strategies for different regions and cities of different sizes within developing countries, thus promoting green technology innovation and green economic development.

The rest of the paper is organized as follows: Section 2 provides a theoretical analysis and posits some hypotheses. Section 3 introduces the data and methodology used in this paper. Section 4 presents the empirical results. Section 5 discusses our findings and makes some suggestions. Section 6 draws conclusions and the shortcomings of the study.

## 2. Theories and Hypotheses

### 2.1. Direct Effects of FER and IER

Both FER and IER essentially put pressure on enterprises to take measures to reduce pollutant emissions. The effect of environmental regulation is influenced by a combination of factors, such as the cost of pollution control, the income status of enterprises, and the difficulty of technological innovation. Regarding the effect of ER, the Compliance Cost Hypothesis focuses on a static cost perspective, while the Porter Hypothesis focuses on dynamic expected benefits. Analysis based on the former is too rigid and one-sided, while analysis based on the latter is too idealistic. In fact, the ultimate effect of city-level environmental regulation depends on the magnitude of the compliance cost effect and the innovation offset effect [42]. An appropriate environmental regulation can motivate enterprises to carry out more innovative activities while their productivity and profits increase accordingly. The result is that a positive innovation offset effect can offset a negative compliance cost effect arising from environmental regulation.

To sum up, the effects of FER and IER on GTI depend on the magnitude of their positive and negative effects. At present, China’s industrialized production mode has shifted from extensive to intensive. China’s national policy is highly focused on ecological civilization and a green economy, and the Five Development Concepts emphasize Innovation and Greenness. With the improvement of environmental legislation and the improvement of standards, it has been difficult for enterprises to meet the requirements of environmental regulations only through end-of-pipe pollution management. Against this background, high-polluting enterprises were forced into technological innovation; they otherwise needed to relocate or to reduce or even stop production. Therefore, at the cities level in China, environmental regulation has a positive impact on GTI. Accordingly, the following hypotheses are posited:

**Hypothesis** **1.** *FER (H1a) and IER (H1b) have a positive impact on GTI in cities.*

### 2.2. The Linkage Effect of FER and IER

FER is a government-led (top-down) mandatory measure to ensure implementation, while IER represents public-led (bottom-up) spontaneous environmental action. Although the implementation subjects and means of FER and IER are quite different, their goals are consistent, and their impact on GTI in cities may be linked. On the one hand, there may be a complementary relationship between FER and IER. The formulation of a series of environmental regulations not only reflects the importance that governments attach to environmental governance issues, but also creates a social atmosphere for environmental governance and improves public awareness of environmental protection. The public’s participation in environmental protection actions creates an atmosphere of national supervision, which can promote local government departments to implement environmental regulation policies. On the other hand, there may also be a substitution relationship between them. FER reflects not only the government’s attitude towards environmental pollution, but also the public’s willingness to improve the ecological environment. At the same time, the public’s attention to, and supervision of, the ecological environment can promote enterprises to actively fulfill their pollution control obligations and force enterprises to carry out green technology innovation. This, to a certain extent, shares the pressure of the government’s environmental regulation responsibilities. When enterprises actively fulfill their environmental governance obligations and carry out green technology innovation, the government will reduce the cost and intensity of supervision. Given the complexity of China’s environmental governance and protection work, forcing the green transformation of enterprises was the joint effect of the government and the public. Moreover, China is a typical “guanxi” society. The relationship between enterprises and the local government is complicated, and the government’s top-down supervision is inevitably not in place, but the public’s bottom-up supervision can compensate for it. Therefore, this paper believes that FER and IER play complementary effects, and the following hypothesis is proposed:

**Hypothesis** **2.** *FER and IER are complementary, and their interactive term, TER, have a positive impact on GTI in cities.*

### 2.3. Heterogeneity of FER and IER Effects

Considering the considerable differences in resource endowments and uneven regional development across China, the role of ER on GTI may be asymmetric. Many empirical studies have found that the effect of ER was not the same in different regions of China [41]. With a high population density, a high intensity of land development, and a high level of industrialization, the eastern coastal regions of China have a rich amount of experience in environmental pollution problems caused by industrial development. As the local governments incorporate the ecological environment into the performance assessment system and public demand for green welfare rises, the government’s supervision and penalties for corporate emissions become stricter, and heavy polluters are forced to transform, shut down, or relocate. In this context, China’s central and western regions have become “havens” for polluting industries [43]. In the process of industrial spatial gradient transfer, the polluting industries were first transferred to the central regions, then to the western region. On the one hand, the level of economic development in the central and western regions is generally low, and the number of industrial enterprises in these regions is very small. Local economic development is, to a certain extent, at the expense of the ecological environment, and for a long time, some areas presented the phenomenon of a “race to the bottom”. Therefore, the governments in the central and western regions may have weak enforcement of environmental regulations and supervision, making it difficult to promote green technology innovation in enterprises. On the other hand, due to the small number of local industrial enterprises and the insufficient experience addressing environmental pollution problems, as well as weak public awareness of environmental protection and an insufficient demand for green welfare, the public’s spontaneous participation in monitoring corporate environmental protection has been weak, making it difficult to put pressure on corporate images. Therefore, the following hypotheses are proposed:

**Hypothesis** **3.** *The impact of FER (H3a), IER (H3b), and TER (H3c) on GTI decreases from the eastern region to the central and western regions.*

There is not only regional heterogeneity in environmental regulations, but also possibly urban heterogeneity. The distribution of industrial enterprises is often dominated by developed large cities, followed by medium cities, and finally small cities. First, in terms of the government’s environmental regulations, large cities have strong demands for green environmental welfare, and the government’s enforcement of environmental regulations is strong. Therefore, when the industrial policy is adjusted, it will put pressure on polluting enterprises and force them to carry out green technology innovation and transformation. In contrast, small cities are more inclined to improve their economic level at the expense of their environment, and few enterprises in small cities may make important contributions to local government tax revenues, economic development, and job creation. Therefore, it is difficult for the governments to impose strict regulatory measures. Second, in terms of the level of innovation in cities, the high level of knowledge stock and technological development in large cities is more favorable to adopting technological innovation in response to environmental regulations. However, small cities are affected by insufficient knowledge stock and innovation ability and are more inclined to choose methods such as strengthening end-to-pipe governance when faced with an increase in environmental intensity, thereby forming a “crowding-out effect” on green technology innovation. Finally, in terms of the public’s environmental monitoring behavior, residents of large cities tend to be more educated on average, and numerous highly qualified residents have a higher demand for environmental protection and green welfare. The public is more motivated to spontaneously monitor corporate environmental protection. On the contrary, the awareness of environmental protection and green environmental welfare consumption in small cities is lower, and the supervision of enterprises’ environmental protection behavior is weak. Therefore, the following hypotheses are proposed:

**Hypothesis** **4.** *The impact of FER (H4a), IER (H4b), and TER (H4c) on GTI decreases as the size of the city decreases.*

## 3. Variables and Methods

### 3.1. Variables and Data

#### 3.1.1. Green Technology Innovation (GTI)

Patents, the main form of intellectual property, have been widely used data in knowledge and innovation research, and the number of patents has been a common indicator of regional innovation capacity [44,45,46,47]. Green patents show two advantages in evaluating the level of GTI in cities: Patents can more accurately reflect the outputs and performance of innovation activities, rather than inputs. The patent’s IPC information can accurately describe the technical field characteristics of innovation activities, which is conducive to the identification of green technology patents.

This study extracted the green patent IPC code from the green patent list published by the World Intellectual Property Organization (WIPO) (https://www.wipo.int/classifications/ipc/en/green_inventory/index.html (accessed on 1 September 2021)). Data are from the China National Intellectual Property Administration (https://www.cnipa.gov.cn/ (accessed on 1 September 2021)), with a limited search period of 2012–2019. The number of green invention patents for each city was obtained, and its logarithm was used to measure GTI.

#### 3.1.2. Environmental Regulation (ER)

Formal Environmental Regulation (FER): In this paper, FER was defined as the degree of government intervention in environmental pollution, which was measured by the discharge of major pollutants corresponding to a unit industrial output value. In order to avoid the bias of results caused by a single index, the comprehensive index of FER was constructed by selecting industrial waste water emissions, industrial sulfur dioxide emissions and industrial smoke emissions corresponding to the unit industrial output value, combined with the availability of data [48,49]. The data are taken from the China Urban Statistical Yearbook. The basic logic is that, to achieve the goal of improving environmental quality, government departments can reduce the amount of pollutants emitted per unit of industrial output in cities by regulating the emission of pollutants from enterprises.

(1) $$FER_{j}=\overset{n}{\underset{i}{\sum }}\alpha_{i}\left(1-\frac{p_{ij}/v_{j}-min\left(p_{ij}/v_{j}\right)}{max\left(p_{ij}/v_{j}\right)-min\left(p_{ij}/v_{j}\right)}\right)$$

where *FER_j_* represents the formal environmental regulation of city *j*. The value is between 0 and 1; the higher the value, the greater the formal environmental regulation intensity. *p_ij_* represents the emission of pollutant *i* in city *j*. *v_j_* represents the industrial output value of city *j*. *n* is 3, which represents the number of pollutant types. *α_i_* represents the weight of pollutant *i*, which is measured by the proportion of pollutant emissions [49,50].

Informal Environmental Regulation (IER): IER is formed by the conscious participation of all sectors of society and citizens in the management of environmental pollution and is a reflection of the public will in the management of environmental protection. Therefore, IER is defined as the degree of spontaneous public participation in environmental pollution management. Nowadays, the Internet has become the main avenue for the public to participate in the management of ecological and environmental pollution, and this “Internet search behavior” is a concentrated expression of the public’s ecological values and environmental protection aspirations. Therefore, IER can be measured by the network attention of the ecological environment pollution. Network attention data comes from the Baidu Index platform (https://index.baidu.com/v2/index.html#/, accessed on 1 September 2021).

Since it is difficult to comprehensively measure IER with a single keyword, this paper considers multiple representative keywords related to “environmental pollution” for comprehensive analysis. However, too many keywords will lead to irrelevant information. The timeliness of indexing will be reduced, and the complexity will be increased. Therefore, after obtaining the initial selection range, the keywords were filtered. Using the Baidu Index automatic recommendation tool, keywords with a high degree of relevance to environmental pollution were searched to eliminate terms that were not included in the Baidu Index. Different keywords were then compared and analyzed for search frequency, and the 21 keywords with the highest search frequency were filtered out according to their ranking. Finally, the daily overall (PC + Mobile) network attention data of Baidu users in each city from 2012 to 2019 were collected, and a total of 300,349,880 pieces of data were collected. The logarithm of the city data was then used to measure the IER.

Dual Environmental Regulation Interaction (TER): In environmental governance and corporate environmental behavior supervision, FER and IER often work together, so it is necessary to study their interaction (TER). In this paper, TER is defined as the degree of joint action of FER and IER, measured by the interaction of FER and IER.

#### 3.1.3. Control Variables

In order to avoid endogeneity problems caused by omitted variables, referring to relevant studies [4,23,41,51], the following control variables were selected:

Human capital level (*hcp*): The level of human capital reflects the quality of a city’s human resources and the human cost of technological innovation in the city. Regions with high levels of human capital tend to have a strong ability to learn and absorb advanced technology and knowledge, so innovative companies tend to cluster in places rich in human capital. This paper uses the number of employees in the tertiary sector to measure the level of urban human capital.

Industrial Development Level (*tvi*): The industrial foundation is a prerequisite for technological innovation, and green technological innovation is modern technology employing the concept of greenness, which is largely an improvement and standard upgrade of traditional industrial technology. Therefore, there is a high correlation between green technology innovation and traditional industrial technology innovation. This paper uses the total industrial output value above the designated size to measure the level of urban industrial development.

Urban consumption level (*con*): The higher the per capita consumption level of urban residents, the stronger their green consumption concept; therefore, the larger the market for green technologies and products, which is conducive to driving enterprises to innovate green technologies and related products. This paper uses the total retail sales of social consumer goods to measure the urban consumption level.

Financial scale of the city (*fin*): Green technology innovation is a high-investment and high-risk production process. The high-level financial development of cities can ease constraints on corporate capital flow and provide risk-sharing and economic incentives for green technology innovation. This paper uses various loan balances of financial institutions at the end of the year to measure the level of urban financial development.

Economic Development Level (*pgdp*): Technological progress is the fundamental source of economic growth, and the level of economic development generally determines the level of capital investment and the accumulation of technological innovation in a country/region or city. Economic development provides a financial guarantee for green technological innovation. This paper uses the per capita GDP to represent the level of economic development.

This paper considered the balanced panel data of 284 cities in China from 2012 to 2019 for empirical analysis. The panel data processing procedure was as follows: First, the average growth method was used to provide the missing data of certain years, and the continuous missing data samples for multiple years were eliminated. Second, in order to reduce the influence of outliers, the data were tailed at the 1% and 99% quantiles. Finally, to eliminate heteroskedasticity, some variables were taken as logarithms (Table 1). The data come from the China Urban Statistical Yearbook and the statistical yearbooks of various provinces and cities over the years. The descriptive statistics of each variable are shown in Table 2. The average value of the logarithm of GTI in the sample is 4.623, the minimum value is 0, the maximum value is 9.670, and the standard deviation is 1.637, which indicates that the number of green invention patents in different cities varies greatly. FER was constructed as a composite index with an average value of 0.321, a minimum value of 0.120, a maximum value of 0.790, and a standard deviation of 0.058, which indicates that government-led FER varies relatively little across cities. By contrast, the average value of the logarithm of the IER is 11.140, the minimum value is 0, and the maximum value is 14.877, with a standard deviation of 1.316, which indicates that public-led IER varies very much across cities. The data description of TER is similar to that of characteristic IER.

### 3.2. Models

#### 3.2.1. OLS

OLS (ordinary least squares) is one of the most basic forms of regression analysis, which is based on the principle of minimizing the sum of squares of the distances from all observations on the scatter plot to the regression line. The advantage of this model is that it requires the fewest conditions and has been widely used in econometrics. This model is suitable for the regression analysis in this study.

(2) $$GTI_{it}=C+\alpha_{1}FER_{it}+\alpha_{2}IER_{it}+\alpha_{3}TER_{it}+\sum_{i=1}^{N}\alpha_{i}ctrl_{it}+u_{i}+\gamma_{t}+\varepsilon_{it}$$

where *GTI_it_* represents the level of green technology innovation in city *i* at year *t*. *FER_it_*, *IER_it_*, and *TER_it_* stand for formal and informal environmental regulation and their interactions, respectively. *i* and *t* represent city and year, respectively, *C* is the intercept term, and *α*_1_, *α*_2_, *α*_3_, and *α_i_* represent undetermined coefficients. *ctrl* represents the set of control variables, and *μ* and *γ* represent the city fixed effect and the time fixed effect, respectively. *ε* represents a random disturbance item.

To avoid model endogeneity issues, all independent variables were lagged by one year. The variance inflation factors (VIFs) were all less than 10, indicating that there was no multicollinearity among the factors. In order to avoid spurious regression, the variables need to be tested for stationarity to ensure the robustness of the estimated results. In this paper, LLC and IPS are used to perform a unit root test on each variable, and the test results show that the *p*-values are all 0.000. As shown in Table 3, the conclusion was that all variables reject the null hypothesis and passed the stationarity test.

#### 3.2.2. GTWR

The GTWR model introduces the time factor based on the GWR model, which can effectively address spatial-temporal non-stationarity and solve the limited problem of cross-sectional data [52,53]. Unlike the traditional spatial econometric model, the GTWR model visualizes the regression coefficients to more intuitively illustrate the spatial differences of variables. In this study, the GTWR model was used to explore the spatial-temporal heterogeneity of environmental regulation on green technology innovation.

(3) $$GTI_{i}=\beta_{0}\left(u_{i},v_{i},t_{i}\right)+\underset{k}{\sum }\beta_{k}(u_{i},v_{i},t_{i})X_{ik}+\varepsilon_{i}$$

where *GTI_i_* represents observations in city *i*. *u_i_* and *v_i_* are the latitude and longitude of city *i*, and *t* is the year. *β_0_*(*u_i_*, *v_i_*, *t_i_*) is the space–time intercept term. *β_k_* (*u_i_*, *v_i_*, *t_i_*) is the regression estimation coefficient of factor *k* in city *i*. *X_ik_* is the value of factor *k* in city *i*. *ε_i_* is the residual.

## 4. Empirical Results

### 4.1. Effects of FER and IER

#### 4.1.1. Direct Effects and Linkage Effects

Table 4 shows the regression results. Mol-1 was used to examine the direct effect of FER on GTI. The results show that FER significantly promoted GTI (0.702, *p* < 0.01). Mol-2 was used to add the squared term of FER to examine the nonlinearity. The results show that the effect of FER was nonlinear, i.e., an inverted U-shape, but the significance was weak (*p* < 0.1). Mol-3 shows that IER also promoted GTI (0.033, *p* < 0.1), and Mol-4 shows that there was no nonlinear effect of IER. These results show that FER and IER both played a positive role on GTI in all cities in China and that H1a and H1b are verified, which supports the Porter Hypothesis. From the size and significance of the model coefficients, the effect of FER was significantly stronger than that of IER. This means that government-led FER has a greater impact on GTI, while public-led IER has a lower impact. Although the effect of FER has been nonlinear, the nonlinear effect is relatively weak and its positive effect dominates. The effect of IER has been weak and does not show nonlinearity, indicating that the impact of IER still has much room for growth. China’s current green technology development and environmental protection still need to be led by the government, and mandatory environmental control measures need to be enforced upon enterprises. At the same time, the public also needs to be guided to participate in environmental governance issues. 

Mol-5 was used to add the interaction term TER, and the results show that TER significantly promoted GTI (0.146, *p* < 0.01). Thus, FER and IER have had a complementary linkage effect, and their combination can promote GTI, which verifies Hypothesis 2. In order to further explore the linkage effect of FER and IER, the model Mol-6 was used to further test the moderating effect. The results show that TER still significantly promoted the level of urban green technology innovation (0.832, *p* < 0.05), which further verifies Hypothesis 2. Government-led FER instruments and public-led IER instruments can work in combination to force enterprises to take actions, such as pollutant emissions reduction and environmental protection, and promote GTI in cities.

#### 4.1.2. The Effects of Control Variables

The coefficients of the control variables are generally stable across the different models, suggesting the robustness of the results. The levels of human capital, industrial development, urban consumption, and urban financial scale all had a positive impact on GTI. However, the levels of human capital and urban consumption were not significant, indicating that the positive role of China’s human capital and consumption on GTI has not fully manifested. This is closely related to the relatively low level of human capital, the relatively weak concept of green consumption, and the low consumption structure of green technology products among residents in China at present. The level of industrial development and financial scale both have had a positive impact on GTI at the 1% significance level. The overall level of China’s industrialization has gradually moved away from the traditional extensive development model of high-energy consumption and high pollution and has shifted to an intensive and efficient development model driven by technology and innovation. Therefore, the level of industrial development has been conducive to the improvement of green technology innovation. Green technology innovation relies on high-investment technology research and development, and the scale of urban finance has provided financial guarantees for high investment in innovation activities, which has been conducive to providing risk-sharing and economic incentives for corporate innovation activities. The level of economic development has had a negative impact on GTI, indicating that China’s current per capita GDP is still low and has not crossed the inflection point of the Environmental Kuznets Curve (EKC), which has not been conducive to GTI.

### 4.2. Heterogeneity of FER and IER Effects

#### 4.2.1. Spatial Heterogeneity

**Figure 1:**
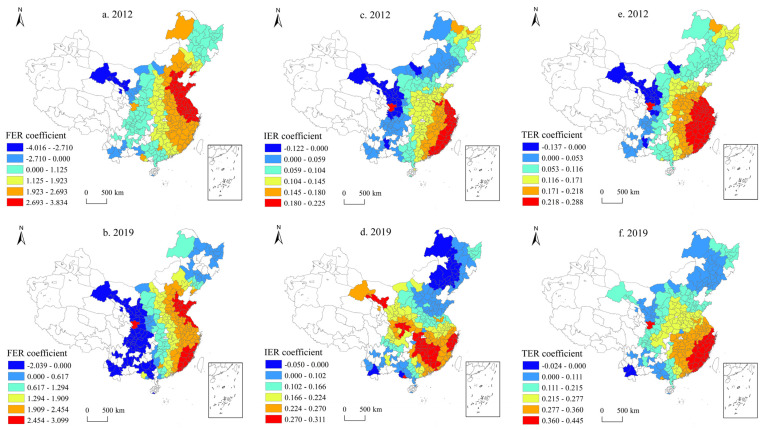
Regression results of GTWR. (**a**) Spatial distribution of FER coefficients in 2012; (**b**) Spatial distribution of FER coefficients in 2019; (**c**) Spatial distribution of IER coefficients in 2012; (**d**) Spatial distribution of IER coefficients in 2019; (**e**) Spatial distribution of TER coefficients in 2012; (**f**) Spatial distribution of TER coefficients in 2019.

When the effect of environmental regulation from all cities of China was examined, regional spatial differences was ignored, so the GTWR was used to examine the spatial heterogeneity (Figure 1). On the whole, obvious regional differences were apparent. The FER coefficient showed an obvious decrease from the positive promotion in the eastern region to the central and western regions, and a negative effect was indicated in a few areas in the west. H3a is verified. The coefficient of FER in 2012 was centered on the concentrated contiguous areas in the Shandong, Jiangsu, and Beijing-Tianjin regions, gradually decreasing toward the central and western periphery, and a negative effect was indicated in a few western cities such as Gansu and Yunnan (Figure 1a). By 2019, the FER coefficient still maintained the east–west trend. The evolution separated some cities in Fujian and their surrounding areas on the southeast coast as the core, and the concentrated and contiguous areas in Gansu, Sichuan, Yunnan, and other places in the west showed a negative impact (Figure 1b). IER showed a core–periphery pattern. In 2012, IER showed a decreasing trend, from the coastal areas of Jiangsu, Shanghai, Zhejiang, and Fujian as the core to the periphery (Figure 1c). By 2019, a part of the core cities was moved to Hunan, Jiangxi and other places, and the coefficients of some cities in Western Inner Mongolia, Liaoning, and Jilin were negative (Figure 1d). The overall results support H3b. TER exhibited a decreasing gradient from the southeast coast to the periphery in both 2012 and 2019, and H3c is verified.

Generally, the influence of FER, IER, and TER on GTI is characterized by a decreasing trend over the east–west gradient and is supplemented by the core–periphery structure. FER, IER, and TER have always had a positive impact in most of the cities in the central-eastern region, which is consistent with the Porter Hypothesis. Environmental regulations have been more robust in the eastern region, and the public’s concept of green environmental protection has been strong. Therefore, in response to the environmental regulations led by the government and the public, enterprises have been under greater pressure for green development and have had a greater impetus for green technology innovation. The effect of environmental regulation in the central and western regions is still weak, especially in a few cities in the western region (such as Gansu, Ningxia and other regions in 2012), which showed a negative effect. These cities have had backward industrial development and funds, and excessive environmental regulation would crowd out R&D funding to the detriment of GTI, which is consistent with the Compliance Cost Hypothesis.

#### 4.2.2. Urban Heterogeneity

GTWR regression was performed to analyze the effect of environmental regulation from the perspective of regional differences, but the model ignored the heterogeneity of city size within each region. There are many cities in China. The differences in the level of economic development of cities of different sizes and their environmental pressures may lead to different implementation effects of environmental regulations. Therefore, 284 cities were further divided into different samples for analysis. This paper refers to the city classification criteria set by the National Development and Reform Commission of China. According to the resident population of urban areas, China’s cities were divided into three categories: small (less than 500,000), medium (500,000 to 1 million), and large (more than 1 million). This division adequately reflects the differences in the hierarchical gradient of Chinese cities in economic and industrial development so that urban heterogeneity can be examined.

The results show (Table 5) that FER, IER, and TER have all had positive effects on cities of all sizes, and all three regression coefficients decrease along with the size of the city, which verifies H4a, H4b, and H4c. Specifically, the regression coefficients of FER in large, medium, and small cities are 1.104 (*p* < 0.01), 0.867 (*p* < 0.05), and 0.540 (*p* > 0.1), respectively, and the regression coefficients of IER in large, medium, and small cities are 0.158 (*p* < 0.01), 0.137 (*p* < 0.05) and 0.018 (*p* > 0.1). It can be seen that there has been a significant city threshold for the effects of FER and IER on GTI, and the strength of the effect has been significantly weaker in small cities especially. The significance of the coefficients decreased with city size; in small cities, the effect of both was not significant. TER has had a positive effect on GTI in cities of different sizes at the 1% significance level. The coefficients only slightly decrease as the size of the city decreases. This suggests that both FER and IER are linked in cities of different sizes, and both can be combined to promote GTI.

Overall, environmental regulation shows clear urban heterogeneity, and the effects of FER, IER, and TER decrease as the size of the city decreases. This means that the larger the city, the stronger the appeal of green technology innovation, and the more prominent the role of environmental regulation. In small cities, due to the small number of enterprises and the lack of local public awareness of environmental protection, although the effects of FER and IER are not shown, the two types of regulation still show some linkage, and their combination does significantly promote GTI.

## 5. Discussion and Policy Implications

### 5.1. Discussion

The world is now facing serious problems of environmental pollution and sustainable development. Promoting green technology innovation through environmental regulation is a key measure in solving these problems. Through theoretical analysis, this study explored the effects of FER and IER on GTI based on OLS and GTWR, which can provide policy insights for the green and sustainable development of cities.

In all Chinese cities, this paper found that FER had a much greater impact on GTI than IER. This finding is consistent with the results of Li et al., who used evolutionary game theory and MATLAB simulations to study the effect of FER and IER on GTI at the enterprise level [54]. This shows that, whether it is from the perspective of cities or enterprises, China is currently dominated by government-led mandatory environmental supervision measures, while the role of IER based on mass environmental supervision is weak. However, the findings of this study are different from the results of Ma et al. and Xu et al., who respectively studied the effects of FER and IER on China’s green energy technology innovation and green technology innovation efficiency [55,56]. This shows that there is industry heterogeneity in the influence of FER and IER on GTI, and there are also differences in the mechanism of green innovation efficiency. In addition, this paper found that FER and IER showed a linkage effect and that they can complement each other to play a positive role on GTI. Wang et al., studying China’s iron and steel industry, reached the same conclusion [57]. This paper thus demonstrates the relationship between China’s FER and IER on GTI based on the overall performance of cities across the country and can act as a reference for coordinating the two environmental regulation strategies.

This paper also found that the effects of FER and IER showed significant regional differences and that the effects of FER and IER decreased from the eastern region to the central and western regions. Similarly, Zhang et al. [58] and Hu et al. [59] showed that there is regional heterogeneity in the effect of environmental regulation on GTI. Shao et al. [60] showed that environmental regulation has a positive effect on GTI in eastern China but not in the central and western regions. However, Nie et al. [61] showed that voluntary environmental regulation has a significant positive effect on green technology innovation regardless of regional factors. Regional heterogeneity in the effect of environmental regulation on GTI was shown to be a common law, but there are differences in the conclusions due to a different research design. In this paper, the GTWR model was used to study the effects of FER and IER on GTI, and the findings show that the impact of FER, IER, and TER on GTI decreases regularly from the eastern region to the central and western regions. The findings expand the spatial dimension of the effect of ER on GTI and can provide a decision-making reference for regionally differentiated environmental regulation.

In view of the significant differences in the size of Chinese cities, this paper investigated urban heterogeneity by dividing cities by size (large, medium, and small), and found that FER and IER have different mechanisms in cities of different sizes. In small cities especially, the impact of environmental regulation is relatively weak. Zhang et al. [62] found that environmental regulations have different effects on GTI in China’s resource-based and non-resource-based cities. Shangguan et al. [63,64] found that there is heterogeneity in the impact of environmental regulation on air pollution and high-quality economic development in large, medium, and small cities. It can be seen that the urban heterogeneity of the impact of environmental regulation is widespread. However, there are relatively few recent studies on the urban heterogeneity of the impact of environmental regulation on GTI. The findings of this study further elucidate the relationship between ER and GTI from the perspective of urban heterogeneity, and provide evidence for the joint formulation of cooperative and coordinated environmental regulation measures by cities of different scales.

### 5.2. Policy Implications

The analysis of the relationship between FER, IER, and GTI in this paper has many policy implications:

First, in the process of the innovative development and green transformation of the city, developing countries such as China should continue to maintain a strategy of government-led mandatory environmental regulation measures as the main means. The government should increase its attention to, and investment in, environmental governance, strengthen the supervision of environmental governance, and establish well-organized and effective environmental governance system standards and norms. The government performance appraisal system should appropriately increase the proportion of environmental governance performance to achieve the goals of environmental protection.

Second, IER formed by public spontaneous supervision should be used as a supplementary means. Through the Internet and other channels, information such as enterprises’ illegal pollution discharge and green innovation should be reported in a timely manner to ensure that the public becomes aware of them. The media should be used to promote the concept of green development, enhance public concern about environmental issues, stimulate the public’s enthusiasm and initiative to participate in environmental governance, implement monitoring functions, and solidify environmental values. The government should increase its support for non-governmental environmental protection organizations and actively guide them to participate in the formulation of environmental management policies.

Third, in China, there are obvious regional differences in economic development and industrialization, government enforcement of environmental protection supervision, and public awareness of environmental protection participation. Therefore, in the process of implementing environmental regulatory measures and guiding the public to participate in environmental governance, a “one-size-fits-all” approach should be avoided, and a regionally differentiated strategy should be adopted. For cities with a backward industrial development, it is necessary to adopt appropriate environmental regulation and pollution discharge fee standards, increase support for those enterprises, and increase their power to innovate green technology, thereby improving the feasibility of green technology innovation among those enterprises.

Finally, in the process of promoting green development through environmental regulation, the relationship between large, medium, and small cities should be coordinated to avoid disorderly competitive development among them. Policies should adopt a coordinated development approach to promote the orderly distribution and transfer of enterprises among large, medium, and small cities. Polluting enterprises should also be prevented from moving to areas with lenient environmental policies, thus causing an environmental sanctuary effect.

## 6. Conclusions and Limitations

### 6.1. Conclusions

This paper puts forward hypotheses on the basis of theoretical analysis and then tests them with empirical research. The number of green technology innovation patents was used to measure GTI in cities. FER was measured by constructing an index of major pollutant emissions per unit of industrial output. IER was measured by the network attention of keywords related to “environmental pollution”. The effect of FER and IER on GTI was then studied. The following conclusions can be made:

(1) In all Chinese cities, both FER and IER have had a positive impact on GTI, and the role of FER has been much stronger than that of IER. TER has had a positive impact on GTI in cities, indicating that FER and IER are linked and show complementarity. The results verify the Porter Hypothesis. China’s current environmental regulation still needs government-led mandatory control measures as the primary strategy, while public-led IER needs to be guided and strengthened.
(2) In terms of spatial heterogeneity, the spatial characteristics of FER, IER, and TER have been dominated by a decreasing trend over the east–west gradient and supplemented by a core–periphery structure. Most cities in the central and eastern regions have had a positive effect, which conforms to the Porter Hypothesis. A very small number of cities in the west have had a negative effect, which is in line with the Compliance Cost Hypothesis. The spatial heterogeneity of environmental regulation effects is widespread, suggesting that environmental regulation in the eastern, central and western regions needs to be tailored to local conditions.
(3) In terms of urban heterogeneity, the effects of FER, IER, and TER all decreased as the size of the city decreased. The coefficients and significance levels of the model regression show that there is an obvious urban threshold for the effects of FER and IER, especially in small cities. This shows that large cities and medium-sized cities have been the main areas where environmental regulation promotes technological innovation, while small cities have been comparatively weak. However, the linkage of FER and IER can play a role in small cities.

### 6.2. Limitations and Future Prospects

This paper focused on the effects of FER and IER on GTI. The main deficiencies are as follows: First, IER was measured by the Baidu index of network attention, but data before 2012 were not available, so this paper’s conclusions about IER are based on data from a short period of time. Thus, the temporal heterogeneity of environmental regulations has not been carefully studied. In addition, it may be too simple to rely solely on network attention to measure IER, and IER needs to be measured from a more comprehensive and multidimensional perspective. Second, this paper is relatively weak in that it uses only one indicator (the number of green technology patents) to measure GTI in cities. A richer and more comprehensive evaluation system for GTI needs to be established. Furthermore, further attempts to use a more comprehensive data measure of FER are necessary. Finally, the environmental regulatory behavior of the government and the public itself can be influenced by many factors. The influence model constructed in this paper considers the contrasting and mutual relationships between the government and the public in promoting urban green technology innovation, but does not consider enterprises an important participant. One future research direction could involve the construction of an evolutionary model that includes the interaction and influence governments, the public, and enterprises.

## Figures and Tables

**Table 1:** Definitions of variables.

Variables	Indicators	Abbreviation	Definition
Dependent variable	Green Technology Innovation	*GTI*	the logarithm of the green invention patents
Core variables	Formal Environmental Regulation	*FER*	according to Formula (1)
	Informal Environmental Regulation	*IER*	the logarithm of the Baidu Index of 21 keywords
	Dual Environmental Regulation Interaction	*TER*	the interaction term of FER and IER
Control variables	Human capital level	*hcp*	the logarithm of employees in the tertiary sector
	Industrial Development Level	*tvi*	the logarithm of the total industrial output value above the designated size
	Urban consumption level	*con*	the logarithm of the total retail sales of social consumer goods
	Financial scale of the city	*fin*	the logarithm of the loan balance of financial institutions at the end of the year
	Economic Development Level	*pgdp*	the logarithm of GDP per capita

**Table 2:** Descriptive statistics of variables.

Variables	Obs	Mean	Std.Dev	Min	Max
*GTI*	2272	4.623	1.637	0.000	9.670
*FER*	2272	0.321	0.058	0.120	0.790
*IER*	2272	11.140	1.316	0.000	14.877
*TER*	2272	9.990	1.323	0.000	9.670
*hcp*	2272	12.197	0.809	9.610	15.730
*tvi*	2272	16.894	1.173	6.820	19.690
*con*	2272	15.629	1.047	5.470	18.880
*fin*	2272	16.822	1.050	14.320	21.050
*pgdp*	2272	1.404	0.775	−1.670	3.970

**Table 3:** Panel unit root test results.

Variables	LLC		IPS		Conclusion
	Statistics	*p*-Value	Statistics	*p*-Value	
*GTI*	−26.477	0.000	−11.072	0.000	Stable
*FER*	−25.620	0.000	−9.311	0.000	Stable
*IER*	−19.511	0.000	−8.788	0.000	Stable
*TER*	−25.782	0.000	−8.867	0.000	Stable
*hcp*	−26.380	0.000	−6.612	0.000	Stable
*tvi*	−77.313	0.000	−5.197	0.000	Stable
*con*	−15.086	0.000	−8.036	0.000	Stable
*fin*	−16.316	0.000	−4.178	0.000	Stable
*pgdp*	−6.190	0.000	−11.693	0.000	Stable

**Table 4:** Regression results for all cities.

Variables	Mol-1	Mol-2	Mol-3	Mol-4	Mol-5	Mol-6
*FER*	0.702 *** (0.249)	3.537 ** (1.734)				−2.337 * (1.437)
*FER^2^*		−5.026 * (3.043)				
*IER*			0.033 * (0.017)	−0.014 (0.043)		−0.702 * (0.382)
*IER^2^*				0.004 (0.003)		
*TER*					0.146 *** (0.029)	0.832 ** (0.382)
*hcp*	0.094 (0.067)	0.091 (0.067)	0.096 (0.067)	0.094 (0.067)	0.094 (0.067)	0.089 (0.067)
*tvi*	0.230 *** (0.043)	0.230 *** (0.043)	0.218 *** (0.043)	0.214 *** (0.043)	0.206 *** (0.042)	0.210 *** (0.042)
*con*	0.047 (0.033)	0.047 (0.033)	0.048 (0.033)	0.048 (0.033)	0.046 (0.033)	0.046 (0.033)
*fin*	0.188 *** (0.069)	0.187 *** (0.069)	0.208 *** (0.069)	0.204 *** (0.069)	0.183 *** (0.068)	0.176 *** (0.068)
*pgdp*	−0.096 *** (0.036)	−0.097 *** (0.036)	−0.088 ** (0.036)	−0.088 ** (0.036)	−0.090 ** (0.036)	−0.093 ** (0.036)
*_cons*	−5.913 *** (1.289)	−6.242 *** (1.303)	−6.117 *** (1.293)	−5.869 *** (1.310)	−6.38 *** (1.281)	−4.61 *** (1.584)
*Adj R²*	0.960	0.966	0.959	0.966	0.960	0.960
*N*	2272	2272	2272	2272	2272	2272

Note: *, *p* < 0.1; **, *p* < 0.05; ***, *p* < 0.01. Robust standard errors in parentheses.

**Table 5:** Grouping regression results of the large, medium and small cities.

Variables	Large City	Medium City	Small City	Large City	Medium City	Small City	Large City	Medium City	Small City
*FER*	1.104 *** (0.354)	0.867 ** (0.375)	0.540 (0.462)						
*IER*				0.158 *** (0.056)	0.137 ** (0.064)	0.018 (0.024)			
*TER*							0.193 *** (0.049)	0.167 *** (0.054)	0.138 *** (0.047)
*ctrl*	Yes	Yes	Yes	Yes	Yes	Yes	Yes	Yes	Yes
*_cons*	−4.673	−2.888	−6.268 **	−5.652 *	−4.281	−6.616 **	−5.825 *	−3.777	−6.688 ***
	(3.157)	(2.665)	(2.666)	(3.206)	(2.694)	(2.658)	(3.164)	(2.650)	(2.642)
*Adj-R^2^*	0.982	0.941	0.880	0.982	0.941	0.880	0.982	0.941	0.881
*N*	680	712	880	680	712	880	680	712	880

Note: *, *p* < 0.1; **, *p* < 0.05; ***, *p* < 0.01. Robust standard errors in parentheses.

## Data Availability

Source of the green patent list: https://www.wipo.int/classifications/ipc/en/green_inventory/index.html. Source of the China National Intellectual Property Administration: https://www.cnipa.gov.cn/. Source of the Baidu Index platform: https://index.baidu.com/v2/index.html#/ (accessed on 1 September 2021).
